# Investigation of Hemp and Flax Fiber-Reinforced EcoPoxy Matrix Biocomposites: Morphological, Mechanical, and Hydrophilic Properties

**DOI:** 10.3390/polym14214530

**Published:** 2022-10-26

**Authors:** Ayyappa Atmakuri, Arvydas Palevicius, Giedrius Janusas, Justas Eimontas

**Affiliations:** 1 Faculty of Mechanical Engineering and Design, Kaunas University of Technology, Studuntu 56, 51424 Kaunas, Lithuania; 2 Lithuanian Energy Institute, Laboratory of Combustion Processes, Breslaujos 3, 44403 Kaunas, Lithuania

**Keywords:** hemp and flax fibers, ecopoxy, mechanical properties, FTIR, morphology, SEM−EDX spectrometer

## Abstract

Modern day industries are highly focused on the development of bio-inspired hybrid natural fiber composites for lightweight biosensor chips, automobile, and microfluidic applications. In the present research, the mechanical properties and morphological characteristics of alkaline (NaOH)-treated hemp, flax, noil hemp, and noil flax fiber-reinforced ecopoxy biocomposites were investigated. The samples were fabricated by employing the hand layup technique followed by the compression molding process. A total of two sets of composites with various weight fractions were fabricated. The samples were tested for mechanical properties such as flexural strength, interlaminar shear strength, moisture absorption, and contact angle measurement. The treated fibers were analyzed by using an optical microscope and Fourier transform infrared spectrometer (FTIR). The morphological characteristics, such as porosity and fracture mechanisms, were investigated by using scanning electron microscopy and SEM−EDX spectroscopy. The results revealed that the flexural properties of hybrid composites vary from 22.62 MPa to 30.04 MPa for hemp and flax fibers and 21.86 MPa to 24.70 MPa for noil fibers, whereas in individual fiber composites, the strength varies from 17.11 MPa to 21.54 MPa for hemp and flax fibers and 15.83 MPa to 18.79 MPa for noil fibers. A similar trend was observed in interlaminar shear properties in both cases. From moisture analysis, the rate of absorption is increased with time up to 144 h and remains constant in both cases. The moisture gain was observed more in individual composites than hybrid composites in both cases. Hence, the impact of hybridization was observed clearly in both cases. Also, hybrid composites showed improved properties compared to individual fiber composites.

## 1. Introduction

In recent years, multiple efforts have been made to produce new, creative, and environmentally friendly materials for diverse purposes due to increasing ecological and environmental consciousness [1]. Natural fibers are being investigated as reinforcements in polymer composites due to the expensive cost of synthetic fibers and their dangerous environmental impact. Natural fiber-based composites offer an environmental benefit by reducing reliance on petroleum-based resources in today’s technologies [2,3]. Biocomposites are composites made up of natural fibers including hemp, flax, sisal, Carlota, banana, pineapple, palm, kenaf, bamboo, jute, coir, and sugarcane combined with biodegradable or non-biodegradable polymers [4,5]. Many researchers are paying attention to the natural fiber-reinforced composites due to their abundance, cost-effectiveness, eco-friendly nature, and lightweight applications. Further applications of natural fiber composites are automobile industries, marine, sports equipment, construction, aerospace, microfluidic [6,7,8], and energy harvesting applications [9].

Hemp and flax fibers are renewable resources with a promising mechanical property that can be used as a reinforcement in composites in a wide range of applications. Many research studies have been conducted to determine the effect of the fiber content and orientation of fibers on the mechanical properties of various natural fiber-reinforced polymer (thermoplastic and thermosetting) composites [10,11,12,13,14]. The importance of fabrication techniques on composite materials has also been studied [15]. In general, increasing the fiber content enhances the flexural strength, torsional strength, and tensile strength of natural fiber composites up to a point, after which they rapidly decline. However, due to weak interfacial adhesion between the reinforcement and matrix material, the composite mechanical properties may be significantly below their optimum values. Therefore, the researchers are finding ways to improve the bonding between the reinforcement and matrix material by employing various chemical treatment techniques [16,17]. The chemical treatments can modify the interface by interacting with both fiber and resin materials on the composite surface. Among the studied chemical treatment processes, alkaline treatment is the most commonly used coupling agent to boost the bonding between the fiber and matrix material [18].

The hybridization of composites influences the advancement of material properties and this has been proven in recent years. Many studies have been focusing on improving the material properties by creating new combinations such as joining two different natural fibers, joining natural fibers with synthetic fiber, and joining two different synthetic fibers in the same matrix material. These composites have been investigated in order to ensure the improvement of mechanical properties over single fiber composites [19,20]. There are several types of hybrid composites that exist by varying the size, filler material, and orientation of fibers. The hybridization may overcome the drawbacks of one component by including another type of fiber material. In general, the hybrid effect varies with the fiber’s mechanical and chemical properties; thus, the effect is less when compared to the individual fiber composites [21,22]. Hybrid reinforcement with decent fiber selection is possible to provide striking properties and meet the present requirement for thermosetting matrix composites [23,24]. In the last few decades, many studies on the hybridization of synthetic fibers and plant (leaf, bast, seed) fibers have been investigated. However, natural fiber-reinforced hybrid composites are less investigated, and the recent studies mainly focused on the combination of natural fiber with artificial fiber. The best example of such composites includes the use of carbon, glass, and aramid in combination with hemp, flax, banana, and pineapple [25,26]. However, with the increasing use of synthetic polymer materials around the world, environmental problems such as waste treatment, waste transfer management, and burning contamination are becoming increasingly important. It shows the adverse effect on environmental and climate change issues [27].

Though there are many advantages to natural fiber hybrid composites, there are some drawbacks, such as the durability and moisture absorption behavior weakening the strength of the composite material. The endurance of the natural fiber composites at the structural lamination scale for their use in load-bearing applications is still unknown. While flax and jute composite columns have been shown in recent studies by M.R. Bambach [28] to have theoretically appropriate structural qualities for light, primary structural applications, such as residential and light commercial structures, flax and jute composite columns are not yet widely used. These tests showed that the start of compression buckling of the narrow cross-sectional elements and the post-buckling redistribution of stresses that result in the final limit state (material failure) dominate structural behavior. The presence of moisture in composite material leads to a weakening of the mechanical performance. Ramakrishnan et al. [29] studied the effect of moisture content on natural fiber-reinforced polymer composites. The experimental results stated that the rate of moisture absorption is less in hybrid composites than in individual fiber composites.

Many researchers were interested in working with natural fibers due to their abundance and ease of usage. Ramesh et al. [30] investigated the mechanical and thermal properties of banana−pineapple fiber-reinforced epoxy composites. The results stated that the hybrid composites made up of banana and pineapple have shown improved mechanical properties and also stated that they can be extended to light load conditions for automotive and construction applications. The flame retardancy properties were analyzed by using thermogravimetric analysis but the results were not up to the mark, which can be achieved by using a proper chemical treatment process. Zalinawati et al. [31] studied the effect of fiber treatment on the mechanical properties of burl palm fiber epoxy composites. In their studies, they treated the burl palm fiber with sodium hydroxide solution and then conducted the fabrication process by using a hand layup technique. The results are stated that the treated fiber composites showed superior mechanical properties to the untreated fiber composites. It was observed that thickness swelling is greater in untreated fiber composites due to greater absorption of water when it is exposed to the wet medium. Torres-Arellano et al. [32] worked on the mechanical properties of natural fiber-reinforced bio-based epoxy resins. This work dealt with the natural fiber-reinforced bio-based epoxy resin composites. The composites were fabricated by using a vacuum-assisted resin infusion process. The results showed that the presence of bio-based epoxy resins in the composite material enhanced the mechanical properties and among all the fibers, the jute fibers showed superior properties to the others. Jamshaid et al. [33] investigated the mechanical and electrical properties of jute, sisal, coconut, and sugarcane natural fiber-reinforced bisphenol F epoxy-based biocomposites. The results stated that among all the natural fiber composites, the sisal/jute hybrid composites exhibited superior mechanical properties compared to the coconut/sugarcane composites. The results from the fabricated samples demonstrate that natural fiber biocomposites could open a new path for their application in electrical goods and as an environmentally friendly alternative. Rajak et al. [34] studied the flexural mechanical properties of natural fiber-reinforced polymer composites and their applications. In this overview, the authors tried to explain the advantage of natural fiber-reinforced composites and their various applications. Terwadkar et al. [35] investigated the mechanical properties of banana and kenaf fiber-reinforced epoxy hybrid composites. In this paper, the effect of hybridization was evaluated by comparing the treated fibers and untreated fibers. The alkaline treatment showed a significant impact on the mechanical properties and hybrid composites showed superior properties. Neves et al. [36] drew the comparison between the biocomposites of epoxy and polyester composites. An attempt has been made on hemp fiber composites by varying their weight percentages such as 10, 20, and 30%. It was observed that the increase in mechanical properties increased the fiber content to some extent. Epoxy-based biocomposites exhibited improved mechanical properties compared to polyester composites. Senthil et al. [37] investigated the mechanical properties of hemp and sisal fiber-based biocomposites. The experimental results specify that hybrid composites displayed a small variation in mechanical properties when the stacking sequence was altered. The hybrid composite with the intercalated arrangement showed the highest tensile modulus when compared with the other hybrid counterparts. Typical failure characteristics of the short beam test, such as inter-laminar shear cracks in the transverse direction, micro-buckling, and fiber rupture, were also observed. It clearly states the lack of chemical treatment of the reinforcements. Bazan et al. [38] worked on bio-based polyethylene composites with flax, basalt, and coconut fibers in the presence of wood flour particles. Results indicated the positive impact on biopolyethylene composites. Among these natural fibers, basalt fiber composites exhibited the greatest strengthening properties. By reviewing these literature studies, we can conclude that natural fibers, which are alkaline-treated, exhibited better mechanical properties by improving the adhesion between the reinforcement and matrix material. Though epoxy resin materials are easy to use (flexible) in composite fabrication, there are a few drawbacks to it such as nonbiodegradability, chemical haggardness, and also not being skin-friendly. To overcome these problems, an attempt has been made in the present research based on the bio-inspired resin materials.

This research work presents the new composites based on ecopoxy (bio epoxy) with hemp, flax, noil hemp, and noil flax natural fibers. A total of two sets (Hemp/Flax, Noil hemp/Noil flax) of hybrid composites with various weight fractions were developed based on the hybridization concept. The composites were fabricated by using the hand layup technique, followed by the compression molding technique. These composites were tested for material properties such as contact angle, moisture absorption, and mechanical properties. The failure mechanism was studied by using a scanning electron microscope. The surface properties were investigated by using the optical microscope. The mechanical properties were investigated and then compared. Also, we aimed to understand whether noil fibers (primary waste after processing) are able to serve as a replacement for the normal fibers. Thus, the obtained results showed the significance of appropriately constituted hybrid biocomposites with natural fibers in the presence of ecopoxy matrix material, holding good quality, decent mechanical, adhesion, and surface properties.

### Research Significance

Due to the increased need for engineering applications of thermoplastic and thermoset polymer materials that outperform metals in a variety of applications, hybrid biocomposites with enhanced characteristics have emerged through the addition of diverse reinforcements. The significance of natural, inexpensive, and abundant materials is highlighted by rising environmental regulations and consumer awareness. Due to the tendency of reinforcement and matrix interactions to increase both the flexibility and rigidity of the natural fiber in one step, the hybridization of one natural fiber with another natural fiber is surprisingly favorable. The change in fiber characteristics and polymer composition in biocomposites reinforced with natural fibers showed the most encouraging results. Natural fiber hybrid biocomposites have garnered great attention since their discovery due to their wide variety of properties in food packaging, biomedical devices, automotive industries, and other consumer applications with better thermal, physical, mechanical, optical, and barrier properties. The presence of moisture and the poor surface properties of natural fiber severely affects the mechanical performance of composite material. Hence, the present article is devoted to modifying the hemp and flax natural fiber surface properties by using the alkaline treatment and heat treatment processes. An ecopoxy matrix, along with hardener material, was introduced to fabricate the hybrid composites. The ecopoxy matrix material contains 36% bioactive content (information from the manufacturer), whereas in traditional epoxy, the bioactive content is less than 8%, which is toxic to the human skin as well as the environment. The excess moisture presented in biocomposites was eliminated by using the post-curing heat treatment process. Also, to minimize the waste material during the fiber processing, the current research work planned to understand the material properties by using noil (primary waste) fibers as well as normal fibers. The fabricated samples allowed for mechanical property evaluation, after which the results were compared.

## 2. Materials and Methods

### 2.1. Materials

Hemp (H), flax (F), noil hemp (NH), and noil flax (NF) fibers were used as reinforcements in the fabrication of composites. These fibers (hemp and flax) were extracted manually from the plants. Noil fibers are processed fibers (primary wastage) from the normal fibers. NaOH solution were used for the purpose of chemical treatment, and this was purchased from Sigma Aldrich (St. Louis, MO, USA). The matrix material used here was ecopoxy (bio-inspired resin material having a bioactivity of 36%), along with hardener (acts as a cross-linking agent), which were purchased from Ecopoxy, (Tychy, Poland). The mechanical properties and chemical characteristics of the individual fibers are given in the following Table 1 and the mechanical properties of ecopoxy resin and hardener is given in Table 2.

### 2.2. Extraction and Chemical Treatment of Natural Fibers

One of the most important aspects of the study was the fiber selection and extraction, followed by the chemical treatment. Hemp fibers were extracted from the cannabis sativa plant. Flax fibers were extracted from the bast underneath the exterior of the stem of the flax plant. Noil fibers were obtained from the primary wasted after the processing of raw fibers. All the extracted fibers were cleaned with distilled water and dried to eliminate the moisture after being recovered from the resources. The fibers were then permitted to undergo an alkaline treatment process. This improves the bonding between the matrix and reinforcement. For the chemical treatment process, a 5% NaOH solution was prepared, added to the fibers separately and kept for the heat treatment process (80° temperature for 4 h). Once the heat treatment process was completed, the fibers were cleaned with water again to maintain the pH level in them and allow for drying. The dried fibers were separated using a cotton comb and a hand-sitting process. To maintain uniformity, all the fibers were cut into fine pieces (2–3 mm length).

### 2.3. Materials Weight Percentage

The weight fraction of both fiber and resin material was considered as per the hybridization concept. In the current research work, a total of two sets of hybrid biocomposites were fabricated based on hemp, flax, noil hemp, and noil flax natural fibers. The hybridization states that the composite material contains more than one reinforcement under the same matrix material with a 0.4 wf.%. The theoretical calculations of weight and volume fractions of both the reinforcement and matrix material are given in the following equations [41,42,43].

The weight fraction of the fiber (reinforcement) material is defined as

(1) $$W_{f}=\frac{Weight\;of\;fibers}{Total\;weight}$$

Then, the weight fraction of the resin (matrix) material is defined as

(2) $$W_{m}=\frac{Weight\;of\;matrix}{Total\;weight}$$

Similarly,

(3) $$W_{m}+W_{f}=1$$

The volume fraction of the fiber (reinforcement) material is given as

(4) $$V_{f}=\frac{Volume\;of\;fibers}{Total\;Volume}$$

Then, the volume fraction of the resin (matrix) material is defined as

(5) $$V_{m}=\frac{Volume\;of\;resin}{Total\;Volume}$$

Similar to the previous equation,

(6) $$V_{m}+V_{f}=1$$

It should be noted that one cannot change the weight fraction completely over to the volume fraction or the other way around. The densities of the fiber $\left(\rho_{f}\right)$ and matrix $\left(\rho_{m}\right)$ materials are the definite weight of the fiber and matrix individually. Then the equation can be written as follows:

(7) $$V_{f}=\frac{\frac{W_{f}}{\rho_{f}}}{\frac{W_{f}}{\rho_{f}}+\frac{W_{m}}{\rho_{m}}}$$

From Equation (7), the weight fraction of fiber is written as

(8) $$W_{f}=\frac{V_{f}\rho_{f}}{V_{f}\rho_{f}+V_{m}\rho_{m}}$$

The weight proportions are given in Table 3.

### 2.4. Fabrication of Composites

Hemp/flax and noil hemp/noil flax natural fiber-reinforced hybrid composites were fabricated by using the hand layup technique, followed by a compression molding process. The fiber materials were measured as per the weight fractions mentioned in the previous Table 3 and then placed in a mold material. The molds were covered with non-stick paper to avoid direct contact between the resin and mold material. After placing the fibers in the mold material, the ecopoxy resin solution was poured into it, a roller was applied on the fibers to get the uniform distribution, and then it was compressed with constant weight and left to undergo the curing process. The entire setup lasted for 72 h for the complete curing process. The biocomposite panels were allowed for the post-curing process after fabrication. For the post-curing process, composite panels were placed in an oven at 70 °C for 6 h. After heat treatment, the samples were cut from the panels as per ASTM standards.

### 2.5. Testing Methods

#### 2.5.1. Interlaminar Shear Properties

The interlaminar shear strength of composite material is the failure shear that occurs when a transverse force is applied to it. To measure the shear strength, all the samples were allowed a notch cut 6.4 mm from the center and half of the thickness of the composite samples before testing. Tinus Olsen H10KT (Horsham, PA, USA) was used to test the samples. The dimensions of the tested samples are considered as per ASTM D-3846 standards. The interlaminar shear strength of the fabricated composites is calculated by using the following equation where *P* is the maximum load and *A* is the failure shear area.

(9) $$\sigma_{S}=\frac{P}{A}\mathrm{MPa}$$

#### 2.5.2. Flexural Strength Properties

A three-point bending test was performed on Tinus Olsen H10K to find out the flexural strength of the composites. All samples were tested as per ASTM D-790 standards. The following relation is used to calculate the flexural strength values where *P* is the maximum load, *L* is the span length, *b* is width, and *d* is the thickness of the composite sample:

(10) $$\sigma_{f}=\frac{3PL}{2bd^{2}}\;\mathrm{MPa}$$

The flexural modulus is computed by dividing the change in stress by the corresponding change in strain and is represented by the slope of the initial straight line segment of the stress−strain curve. As a result, the flexural modulus is defined as the ratio of stress to strain. The flexural modulus of composites is calculated by using the following relation where *L* is the length, *m* is the slope of the stress−strain curve, *b* is the width, and *d* is the thickness of the composite.

(11) $$E_{B}=\frac{L^{3}m}{4bd^{3}}\;\mathrm{MPa}$$

#### 2.5.3. Hydrophilic Properties

**Figure 1:**
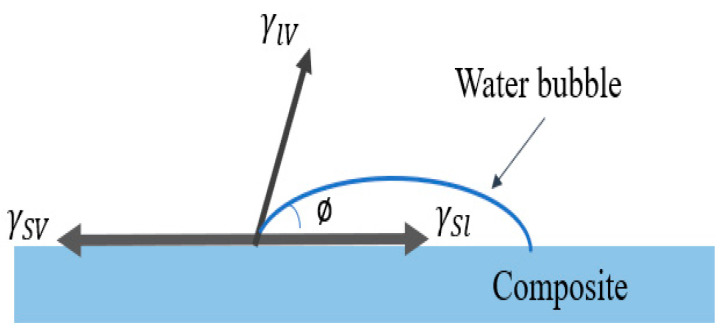
Contact angle formed on composite surface with interfacial tensions of the three phases.

The wettability of the composite surface is measured by using the contact angle measurement analysis. It is also possible to investigate the wetting of and dynamic contact angles on composite samples that are used as the test body. The main objective of this test is to determine the hydrophobicity of the composite material. The schematic representation of the contact angle on the composite surface is shown in Figure 1 and the test specimen standards were considered as per ASTM D-7334 standards.

#### 2.5.4. Moisture Absorption

The rate of absorption of the composite samples was calculated by using the moisture analysis test. It is necessary to find out the rate of absorption when the composite sample is exposed to the moisture content. To evaluate the rate of absorption, all the samples were placed in a glass container full of distilled water. The rate is calculated by measuring the initial weight and final weight (after removal from the water) of the composite samples. The specimen standards were followed from the ASTM D-570. The amount of water absorbed by the composites depends on the percentage of cellulose and the presence of porosity content. The following equation is used to calculate the absorption rate:

(12) $$Rate\;of\;Absorption=\frac{Final\;weight-Initial\;weight}{Initil\;weight}\times 100$$

#### 2.5.5. Optical Microscopic Analysis

Fiber surface modification is often done by physical or chemical means to increase reinforcement−matrix adhesion. This results in a reduction in moisture gain as well as changes in the fiber surface. Changes in the fiber’s surface morphology are critical to address its interaction with the polymer matrix in the composite materials. The effect of chemical treatment on the natural fibers is analyzed by using the optical microscope.

#### 2.5.6. FTIR Analysis

The surface functional group analysis was performed using Fourier transform infrared spectrometer (FTIR, Bruker tensor 27, Borken, Germany). For the FTIR analysis, samples were first dried at 105 °C for 24 h according to the ISO 579 standard. After the preparation, the sample was placed on a horizontal ZnSe crystal plate with a 45° incidence angle and 10 internal reflections, pressed with a fixator to avoid any gap between the plate and sample. A background reference spectra was obtained before every experiment to eliminate the influence of the atmosphere. The scanning time of every wavelength is 32 times, while the wavelength interval is from 650 to 4000 cm^−1^. The resolution of the detector is 4 cm^−1^.

#### 2.5.7. SEM and EDX Analysis

The morphological studies, biocomposite failure mechanisms, and chemical compositions of the biocomposites were investigated by using the SEM (scanning electron microscope) from Hitachi along with an EDX (energy dispersive X-ray) spectrometer from Bruker, Billerica, MA, USA.

## 3. Results and Discussion

### 3.1. Interlaminar Shear Strength Test

The interlaminar shear strength properties of ecopoxy-based natural fiber hybrid biocomposites were investigated and the results are presented in the following graphs. For each sample, a total of five composites were tested and the dimensions of a rectangular cross-section composite were taken as 79.6 × 12.7 × 4.6 mm in length, width, and thickness. Notch depth is taken as half of the specimen’s thickness. All the samples were tested at room temperature and a constant relative humidity (26%) because it was observed from the literature that shear strength decreases when the humidity increases. Ali et al. [44] investigated the mechanical properties of bamboo fiber composites for structural applications. The results stated that the shear strength was decreased when the humidity increased from 60% to 90%.

**Figure 2:**
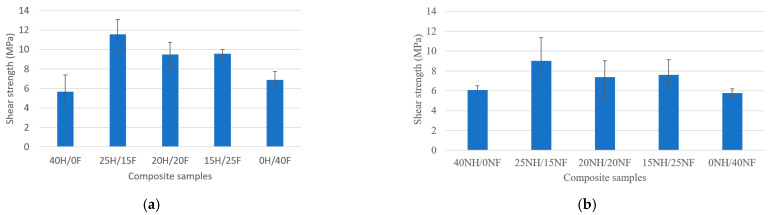
Interlaminar shear strength behavior of (**a**) H/F and (**b**) NH/NF natural fiber hybrid biocomposites.

Figure 2a,b shows the interlaminar shear strength properties of the hemp−flax, noil hemp−noil flax natural fiber hybrid biocomposites. As can be seen from the obtained results, the shear strength values of hybrid biocomposites are superior to the pure fiber (individual fiber) biocomposites in both cases. Natural hemp and flax fiber biocomposites showed better shear strength properties than noil fiber biocomposites. In the first set (H/F) of biocomposites, 25H/15F hybrid biocomposites showed the highest shear strength with 13.10 MPa, and pure hemp (40H/0F) showed the least with 5.67 MPa. To contrast, in the second set (NH/NF) of biocomposites, 25NH/15NF composites showed the highest shear strength with 9.03 MPa and pure noil flax (0NH/40NF) showed the least with 5.43 MPa. Overall, hemp fiber-rich hybrid biocomposites showed superior properties in both cases. The reasons for this can be attributed to the poor bonding between the fiber reinforcement and matrix material in pure composites, which causes an agglomeration, a loss of strength, and possible fabrication faults, whereas in hybrid composites the hybridization impacts the fibers, contributing superior flexural strength to the composites in both circumstances.

### 3.2. Flexural Strength Test

The flexural strength properties of ecopoxy-based natural fiber hybrid biocomposites were investigated and the results are presented in the following graphs. A 3-point bending test was performed on Tinus Olsen at 21° and 28% relative humidity to calculate the bending strength of biocomposites. For each sample, a total of five composites were tested and the dimensions were taken as 80 × 20 × 4.6 mm in length, width, and thickness as per ASTM standards and the samples were taken in the form of a rectangular cross-section.

**Figure 3:**
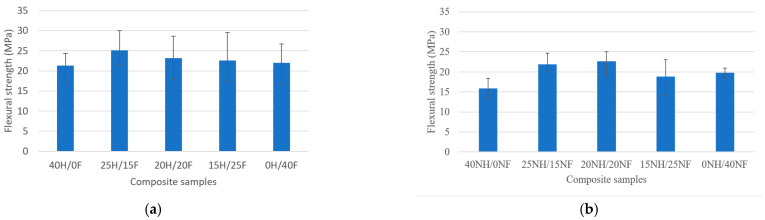
Flexural strength behavior of (**a**) H/F and (**b**) NH/NF natural fiber hybrid biocomposites.

The flexural strength properties of natural fiber hybrid biocomposites are given in Figure 3a,b. In both cases, pure composites showed the least flexural strength values, whereas hybrid composites showed superior properties and minimal variation in the outcomes. Hemp/flax fiber hybrid biocomposites showed a range between 17 MPa and 30 MPa, whereas noil fiber biocomposites exhibited a range between 13 MPa and 24 MPa. This is due to the fiber’s hybridization effect, which gives the biocomposites higher flexural strength in both circumstances. Poor bonding between the reinforcement and resin material in pure biocomposites results in the agglomeration and, hence, a loss of strength and mechanical performance could be the cause of pure biocomposites’ reduced strength. Also, it is evident from the SEM analysis that the presence of internal defects such as matrix cracks, broken fibers, and voids may weaken the strength of a composite material. It was observed from the results that the presence of internal defects is reduced in hybrid composites compared to individual composites in both cases. The hybridization impact was observed clearly in both cases in terms of increasing the strength. A similar trend in the results was witnessed by various researchers investigating the flexural strength of natural fibers [45,46]. In most cases, the flexural strength increases with an increase in the fiber content up to 40%, after which it starts decreasing.

#### Flexural Modulus

**Figure 4:**
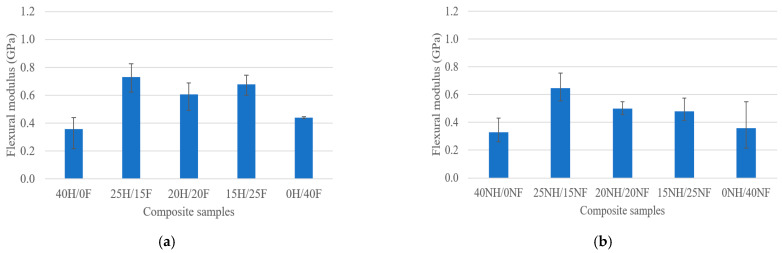
Flexural modulus of (**a**) H/F and (**b**) NH/NF natural fiber hybrid biocomposites.

The results presented in Figure 4a,b show the assessment of the flexural modulus properties of various hybrid fiber biocomposites. The results are followed a similar trend to the previous results. The hemp and flax fiber hybrid biocomposites showed higher flexural modulus properties than noil hemp and noil flax fiber hybrid biocomposites. Pure noil hemp fiber biocomposites showed the least flexural modulus with 0.261 GPa, whereas 25H/15F showed the highest with 0.825 GPa. There is not much variation in the hemp and flax hybrid composites as the values are close to each other, whereas in noil fiber biocomposites, the variation is quite observable.

### 3.3. Contact Angle Measurement Analysis

**Figure 5:**
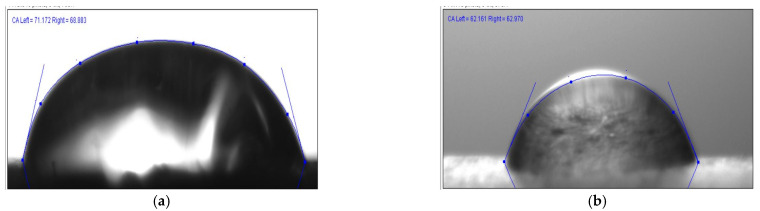
Contact angle image illustration for (**a**) H/F and (**b**) NH/NF biocomposites.

The contact angle measurement illustrations of the biocomposites are shown in Figure 5a,b. The lighting has been meticulously planned to eliminate any light reflections that may detract from the measurement. Precautions were also taken to keep the drips from becoming contaminated by air pollutants such as dust and particulates.

**Figure 6:**
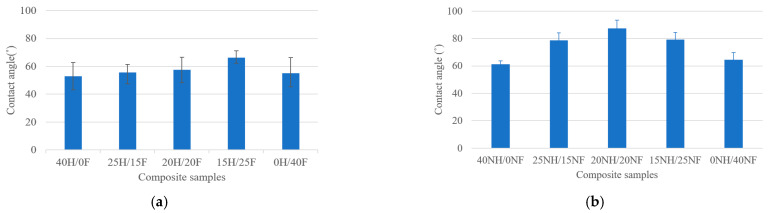
Contact angle measurement for (**a**) H/F and (**b**) NH/NF natural fiber biocomposites.

The graphical representation of measured contact angles for various natural fiber hybrid biocomposites is shown in Figure 6a,b. From the obtained results, the contact angle maximum (87.3°) was observed for the 20NH/20NF hybrid composite and the lowest (53°) was observed for the 40H/0F. Hemp and flax biocomposites showed the contact angle ranging from 53° to 66° and noil fiber composites exhibited angles between 61° and 87°. According to the literature [47,48], a material is well-thought-out as a hydrophilic material if the contact angle is <90° and if it is >90°, it is well-thought-out as a hydrophobic material. This means that all the composites showed hydrophilic surface properties, whereas the 20NH/20NF hybrid fiber composites almost fall in the range of hydrophobic material properties. Furthermore, the contact angle was investigated after 15 s, 30 s, and 45 s for all the biocomposites with distilled water to observe the spreading of water droplets on the biocomposites’ surface. A study decrement was observed for the water contact angle for all biocomposites.

### 3.4. Moisture Analysis

The absorption rate for the fabricated composites was calculated by using the moisture absorption test. The absorption rate was calculated for 7 continuous days and the values were noted for 24 h intervals. All the biocomposites were placed in an oven for 30 min before conducting the test. This test was performed at 18° and 26% humidity in the environment.

**Figure 7:**
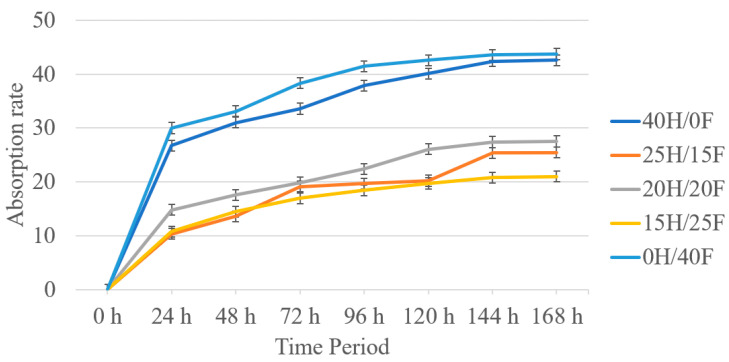
Time period vs. absorption rate for H/F hybrid biocomposites.

**Figure 8:**
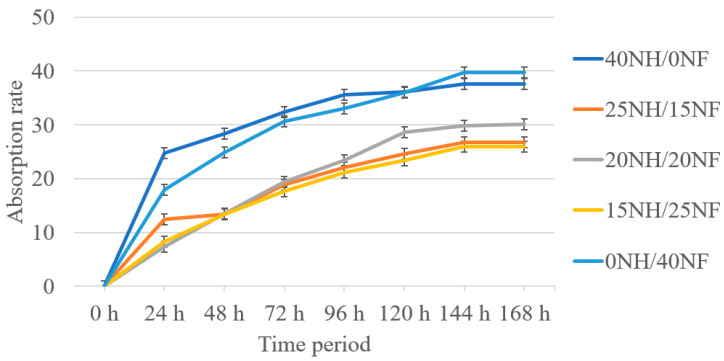
Time period vs. absorption rate for NH/NF hybrid biocomposites.

Figure 7 and Figure 8 below show the absorption rate for hemp/flax and noil hemp/flax fiber hybrid biocomposites. The results state that the absorption rate for the composition increased with time in both cases. A similar trend was observed in both cases. Pure composites showed the highest rate of absorption, whereas hybrid composites showed the lowest rate, with the values being close to each other. The reasons for this can be attributed to the presence of voids (porosity content) in the pure composites and the presence of hydroxyl groups in the pure composites, leading to the attraction of more water content. Overall, hemp and flax fiber biocomposites showed a lower rate of absorption than noil biocomposites.

### 3.5. Fiber Morphology Studies

The fibers’ surface morphology and the effect of chemical treatment on the natural fibers used in this research work are analyzed by using an optical microscope. The following figures differentiate the treated and untreated natural fibers.

**Figure 9:**
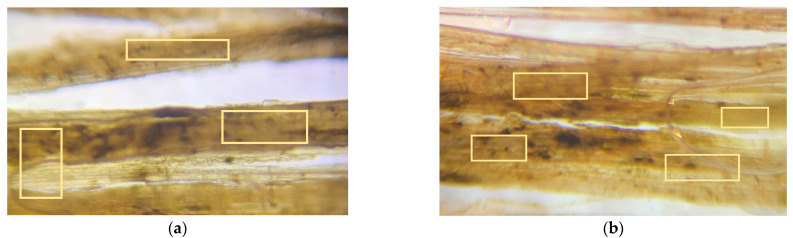
Microscopic images for untreated (**a**) hemp and (**b**) flax fibers, with all having a smooth surface finish.

**Figure 10:**
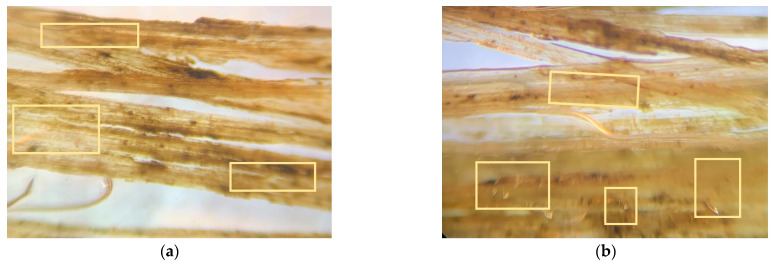
Microscopic images for treated (**a**) hemp and (**b**) flax fibers, with all having a rough surface finish.

The surface morphology of the fibers was studied using an optical microscope. The microscopic images of the fiber surfaces are presented in Figure 9 and Figure 10. Figure 9a,b show the optical microscope images of untreated hemp and flax fibers, which have a smooth surface finish. This is due to the presence of adhesive polysaccharides on the surface of fibers. In contrast, Figure 10a,b show the images of treated hemp and flax fibers, which have a rough surface finish. This is due to the alkaline (NaOH) treatment of natural fibers. The polysaccharides were removed from the surface of the fibers and fibers were also separated from each other. The roughness on the fibers improves the adhesion between the reinforcement and matrix material.

**Figure 11:**
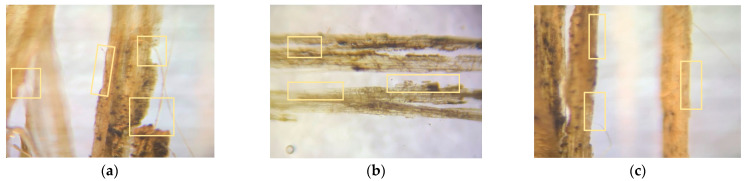

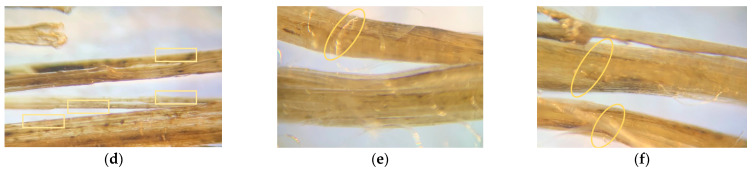
Microscopic images for untreated noil hemp (**a**), noil flax (**b**) fibers with microcracks, treated noil hemp (**c**), noil flax(**d**) fibers without microcracks, untreated noil hemp (**e**) with kink band-treated noil hemp, and (**f**) without kink bands.

The microcracks and kink bands are two forms of flaws that are normally predicted to be found in the cell walls of natural fibers. Figure 11a,b shows the microcracks observed in the optical micrographs of noil hemp and noil flax fibers. However, Figure 11c,d shows the microscopic images for treated noil hemp and noil flax fibers without microcracks in the fiber cell wall. The kink bands were observed in untreated noil hemp fibers in Figure 11e. After chemical treatment, the kink bands were eliminated and shown in Figure 11f. The reinforcement materials act as a load carrier in a composite material. Therefore, the presence of microcracks and kink bands affects the fiber properties and lowers the strength. We can say that to avoid these kinds of defects in natural fibers, a chemical treatment is one of the best solutions.

### 3.6. FTIR Analysis

The fibers’ surface morphology and the influence of chemical treatment on natural fibers used in this research work are analyzed by using the FTIR analysis. The following figures illustrate the treated and untreated natural fibers.

**Figure 12:**
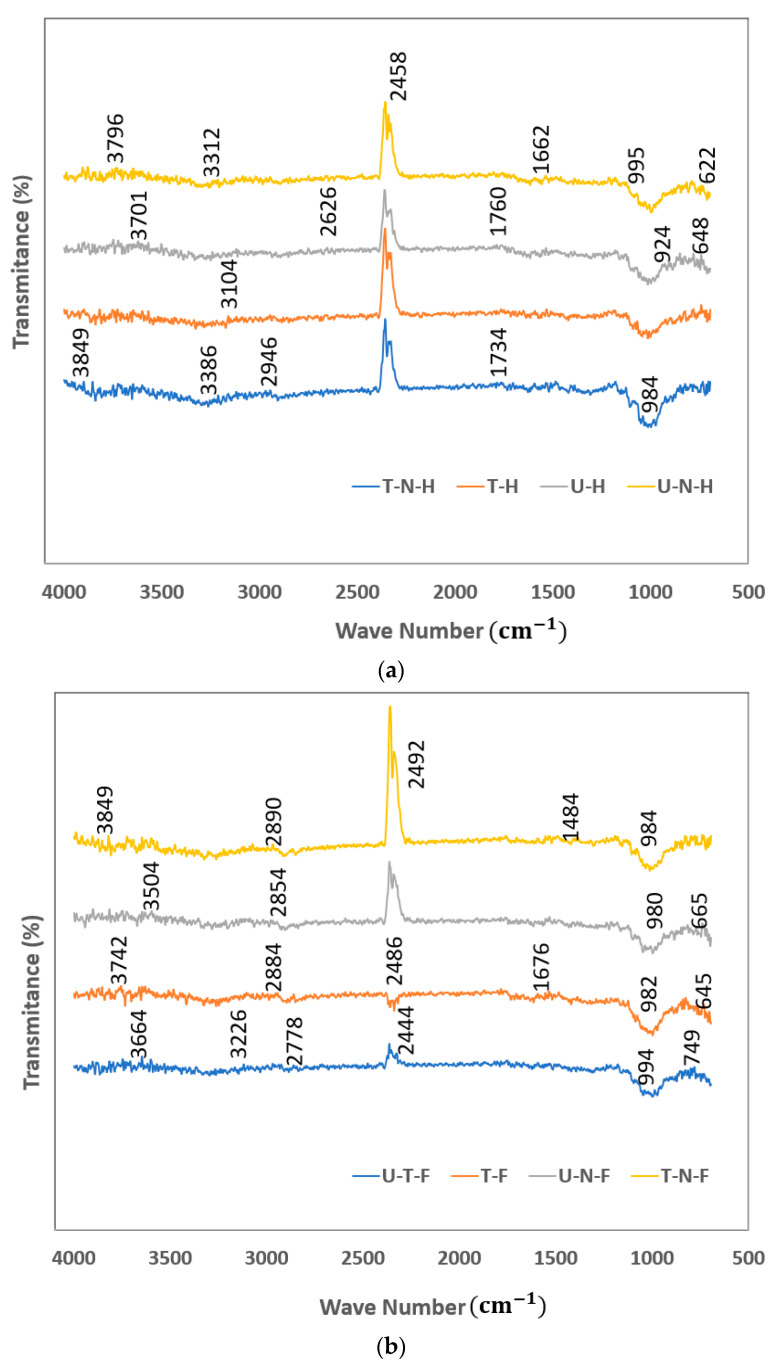
FTIR analysis of treated and untreated hemp and noil hemp fibers (**a**) and treated and untreated flax and noil flax fibers (**b**).

The above Figure 12a,b show the influence of chemical treatment on natural fibers, analyzed by using the Fourier-transformed infrared spectroscopy analysis. The character peaks of treated hemp (T-H), treated noil hemp (T-N-H), untreated hemp (U-H), and untreated noil hemp (U-N-H) fibers are shown in Figure 12a. The character peaks of treated flax (T-F), treated noil flax (T-N-F), untreated flax (U-F), and untreated noil flax (U-N-F) fibers are shown in Figure 12b. These character peaks of fiber materials are attributed to the presence of cellulose, hemicellulose, and lignin content in the fiber material. In general, the IR spectra for natural fibers are presented in the 3250 to 2650 cm^−1^ range and the large bands indicate the deformation of the O-H groups. For the untreated hemp, flax, noil hemp, and noil flax fibers, this peak is found in the range between 3374 and 2846 cm^−1^. The detected absorption band is connected to the O-H group’s deformation. The second large peak, followed by the O-H group, is observed at 2672 cm^−1^, which exhibits the C-O group of the hemicellulose in UH. The characteristic peak at 1584 cm^−1^ is characteristic of the N-H group of cellulose. The peak at 523 cm^−1^ is related to the irregular deformation of C-I of the cellulose and hemicellulose. Whereas in treated fibers, the highest peak is observed around 2780 cm^−1^. The large peak represents the O-H groups in cellulose. The intensity of this peak is higher than the peak observed in the untreated fibers. It was observed from the figure that the obscene C-O band of the carboxyl group is present in the hemicellulose of natural fibers. Which indicates the removal of hemicellulose in the alkaline-treated natural fibers. Also, in the treated fibers, the lignin traces were not absorbed much. Therefore, due to the elimination of hemicellulose and lignin content in the natural fibers, the moisture intake of natural fibers can be reduced, which adversely affects the mechanical properties of the natural fiber composites.

### 3.7. Scanning Electron Microscope (SEM) Analysis

The fracture mechanism of the composite samples was analyzed by using the scanning electron microscope. The SEM images of the hemp/flax and noil hemp/noil flax fiber hybrid biocomposite flexural test specimens were taken to analyze the defects in them.

**Figure 13:**
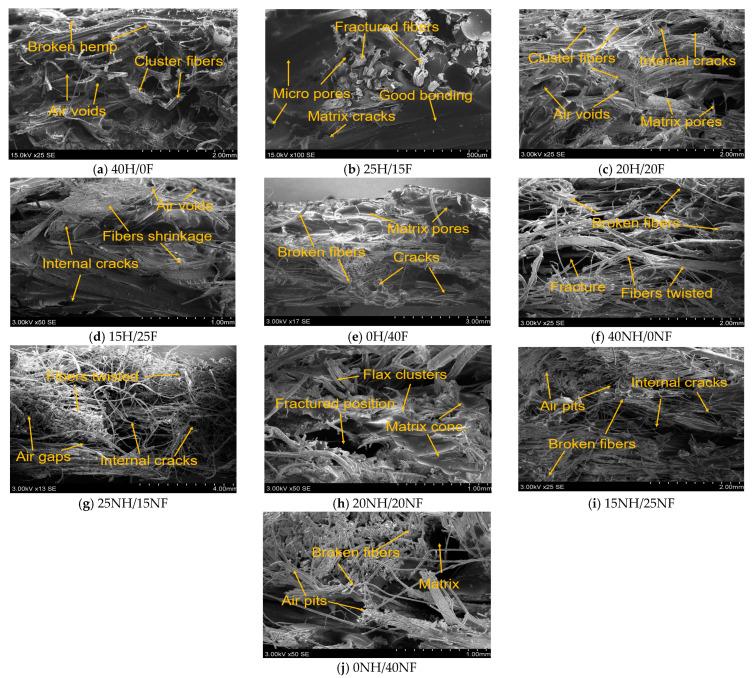
SEM micrographs for hemp/flax fiber biocomposite flexural test fracture samples.

Figure 13a–e above depicts the scanning electron micrographs taken for the fracture flexural test specimens of hemp and flax fiber hybrid biocomposites. From Figure 13a,e, the breakage of fibers was observed in pure biocomposites. The breakage of the fibers lowers the strength of the composites because reinforcements are the load-carrying agent in a composite material when it is subjected to the external loading (force). Also, the presence of microcracks and air voids can be observed in hybrid composites (Figure 13b–d); the presence of defects in the composite material may lower the strength of the composites. The reasons for these defects could be fabrication error, residual stress from the curing process, or poor adhesion between the matrix and reinforcement. Overall, 40H/0F and 0H/40F biocomposites have more defects than hybrid biocomposites, leading to weaker strength. Figure 13f–i shows SEM micrographs of the shattered surfaces of the generated biocomposites (NH/NF) coming from samples that perform well in most of the attributes studied. It was observed from the results that the presence of fiber clusters and twisted fibers in the pure noil biocomposites (Figure 13f,j) may cause the agglomeration of the fibers in the composite material and lower the strength as well. The presence of air pits is observed in Figure 13g–i, which attracts the moisture easily. The reasons for the presence of internal defects are explained earlier.

**Figure 14:**
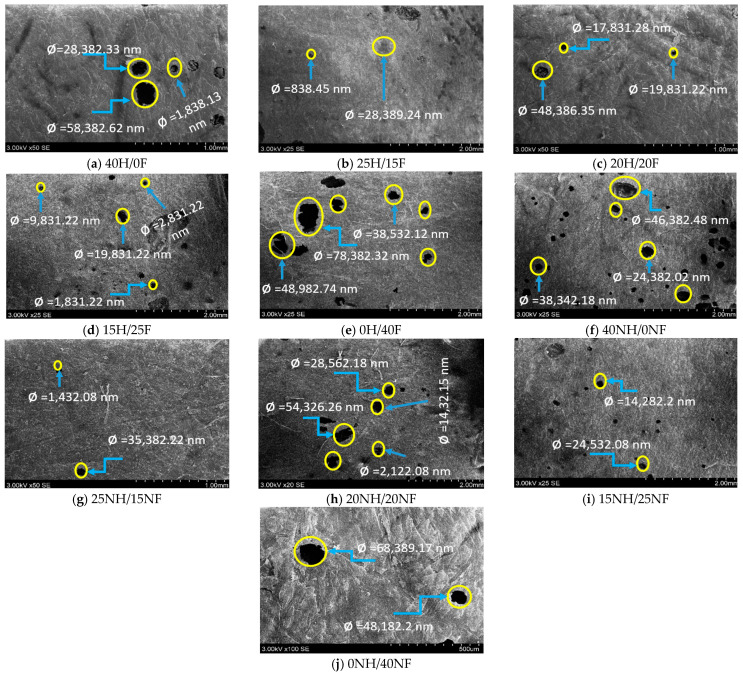
SEM micrographs for porosity content in hemp and flax fiber hybrid biocomposites.

The rate of absorption for the composite material mainly depends on the presence of porosity and the fiber content. Figure 14a–e shows the presence of porosity (air voids) in hemp and flax fiber hybrid composites and f–j shows the noil hemp and noil flax fiber hybrid composites. It was observed that pure biocomposites have a greater void percentage, whereas hybrid composites have a low percentage. The porosity content in 25H/15F (Figure 14b) is minimal so it enhances the improvement in strength. Also, it was observed that 40NH/0NF composites have a greater void percentage and 25NH/15NF fiber composites have the least void content. In both cases, hybrid biocomposites have a lower void percentage, which reflects the improvement in strength, whereas pure biocomposites have more void content. Hence, the rate of absorption is higher in pure composites and lower in hybrid composites in both cases.

### 3.8. SEM−EDX Spectrum Analysis

The chemical composition presented in the natural fiber biocomposites are analyzed with scanning electron microscopic energy dispersive X-ray spectroscopy and the results are shown in the following figures.

**Figure 15:**
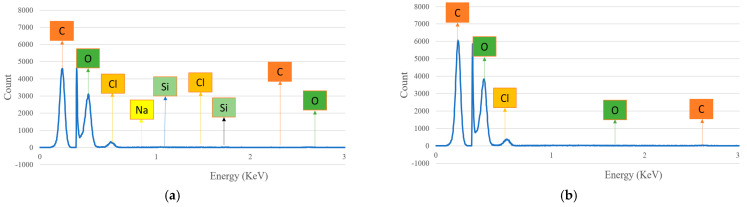
Spectrum analysis of chemical composition for (**a**) hemp and (**b**) flax fiber biocomposites at surface position.

The chemical composition presented in the hemp and flax fiber hybrid biocomposites at surface position is studied by using the SEM−energy dispersive X-Ray spectrometer and the results are described in Figure 15a,b. The percentage of the weight concentration and atomic concentration of the individual elements at the surface position are given in Table 4. From the results, it was observed that there is no such variation in the biocomposites at the surface position. In both fibers, the percentage of carbon takes the top place, followed by oxygen. The presence of sodium and silicon is observed in hemp fiber biocomposites, though the percentage is minimal. This was not observed in the flax fiber composites. The presence of oxygen in the composite surface may form hydroxyl bonds with hydrogen present in the cellulose content, which leads it to attract greater moisture content and weaken the composite’s strength.

After the surface position, the spectrum analysis was carried out at the fractured position to understand the internal composition of the hemp and flax fiber biocomposites. The resultant micrographs are shown in following figures.

**Figure 16:**
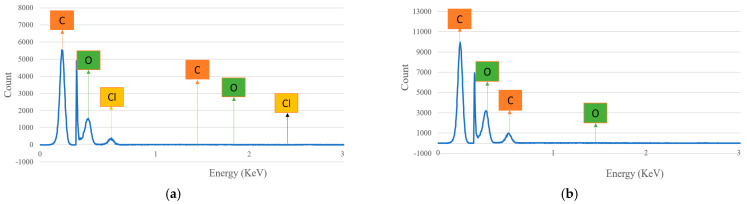
Spectrum analysis of chemical structure for (**a**) hemp and (**b**) flax fiber biocomposites at fractured position.

The chemical composition presented in the hemp and flax fiber hybrid biocomposites at fractured position was studied by using the SEM−EDX spectrometer and the results are described in Figure 16a,b. The percentage of the weight concentration and atomic concentration of the individual elements at the fractured position are given in Table 5. From the results, it was observed that there is some variation in the biocomposites at the fractured position, unlike at the surface. In both fibers, the percentage of carbon takes the top place, followed by oxygen. The presence of chlorine is observed in hemp fiber biocomposites, though the percentage is very small. This was not observed in the flax composites.

After the spectrum acquisition for hemp and flax fiber biocomposites at the surface and fractured positions, we can deduce that the proportion of oxygen is greater at the fractured place than at the surface and it is the opposite for the carbon percentage. The presence of chlorine (Cl), sodium (Na), silicon (Si) along with oxygen (O), and carbon (C) was also observed, albeit in very low percentages. This reflects that the concentration of matrix (ecopoxy) material is greater on the surface than in the fractured position, which leads to a smooth surface finish at the surface of the composite.

## 4. Conclusions

The current research on hemp, flax, noil hemp, noil flax natural fiber-reinforced ecopoxy hybrid biocomposites was fabricated using the hand layup technique, followed by a compression molding process. The fibers were treated with a 5% NaOH solution before fabrication. The fabricated composites underwent mechanical and morphological testing and the results are as follows:

1. Hemp and flax fiber hybrid biocomposites showed superior mechanical properties to noil hemp and noil flax fiber biocomposites. In both cases, hybrid biocomposites showed improved properties compared to pure biocomposites. From the interlaminar shear test results, hemp and flax fiber biocomposites showed a range between 5.67 and 13.10 MPa, whereas noil fibers were from 4.9 to 11.3 MPa. From the flexural test results, the 25H/15F hybrid biocomposites exhibited the maximum flexural strength and modulus as 30.04 MPa and 0.825 GPa and pure noil hemp (40NH/0NF) fiber biocomposites exhibited the minimum as 13.31 MPa and 3.95 GPa.
2. All of the biocomposites had a contact angle of fewer than 90 degrees based on contact angle measurement, indicating that they have hydrophilic surface qualities. The maximum contact angle was observed for 20NH/20NF at 87.31° with the measurement error of ±1.56° and the smallest contact angle was observed for 40H/0F at 53.01° with the measurement error of ±1.94°. From the rate of absorption analysis, all the biocomposites responded to the moisture and the rate increased up to 144 h, remaining constant. In both cases, pure biocomposites absorbed more water than hybrid biocomposites.
3. The fiber morphology studies of treated and untreated fibers were investigated by using the optical microscope. The results were presented in a way that contrasted the treated and untreated fibers in terms of defects. The fracture mechanisms and the porosity content in the biocomposites were investigated by using scanning electron microscopy. The spectra acquisitions for the chemical composition presented in hemp and flax fiber biocomposites were observed by using SEM−EDX spectrographs.

Overall, hemp and flax fiber hybrid biocomposites showed superior properties to noil fiber biocomposites so they can be efficiently used as reinforcements in lightweight biosensor chips, flexible electronics, microfluidics, and biocomposite applications. Although these hybridization techniques and chemical treatment processes achieved promising results, more investigations are essential to improve the porosity content and moisture properties, which may be possible with the addition of filler materials, additive materials, and heat treatment processes.

## Figures and Tables

**Table 1:** Hemp and flax fiber chemical composition and mechanical properties [39,40].

Properties	Hemp	Flax
Cellulose (%)	70–74	64–70
Hemicellulose (%)	21–24	16–18
Lignin (%)	3.7–5.7	2–2.2
Pectin (%)	7.3	2.3
Elongation (%)	1.6–4.0	1.2–1.6
Moisture (%)	6.2–12	8–12
Density (g/cm^3^)	1.48	1.4
Young’s modulus (GPa)	70	60–80
Microfibrillar angle (°)	2–6	5–8
Tensile strength (MPa)	550–900	800–1500
Specific strength (s/g)	393–643	570–1070

**Table 2:** Liquid ecopoxy resin and hardener material properties.

Properties	Ecopoxy	Hardener
Color	Viscous liquid	Clear
Viscosity at 25 °C	800 = 1000 cP	100–200 cP
Specific gravity	1.1	1.0
Bio content	36%	-
Mix ratio	2	1
Gel time at 25 °C	20–25 min	20–25 min
Full cure at 25 °C	72 h	72 h

**Table 3:** Composite weight fractions.

Composites	Hemp Fiber (%)	Flax Fiber (%)	Total Fiber Vol. (%)	Total Resin Vol. (%)
40H/0F	40	0	40	60
25H/15F	25	15	40	60
20H/20F	20	20	40	60
15H/25F	15	25	40	60
0H/40F	0	40	40	60
	Noil Hemp Fiber (%)	Noil Flax Fiber (%)		
40NH/0NF	40	0	40	60
25NH/15NF	25	15	40	60
20NH/20NF	20	20	40	60
15NH/25NF	15	25	40	60
0NH/40NF	0	40	40	60

**Table 4:** Spectrum analysis for chemical configuration of natural fiber hybrid biocomposites at the surface position.

Element Type	Element No.	Atomic Weight	Hemp Biocomposite		Flax Biocomposite	
			Weight (%)	Atomic Conc. (%)	Weight (%)	Atomic Conc. (%)
Carbon (C)	6	12	76.29	81.61	77.17	82.18
Oxygen(O)	8	16	22.04	17.70	21.85	17.47
Chlorine (Cl)	17	35.5	1.03	0.37	0.97	0.35
Sodium (Na)	11	23	0.28	0.16	--	--
Silicon (Si)	14	28	0.35	0.16	--	--

**Table 5:** Spectrum analysis for chemical configuration of natural fiber hybrid biocomposites at the fractured position.

Element Type	Element No.	Atomic Weight	Hemp Biocomposite		Flax Biocomposite	
			Weight (%)	Atomic Conc. (%)	Weight (%)	Atomic Conc. (%)
Carbon (C)	6	12	63.73	70.26	68.82	74.62
Oxygen(O)	8	16	35.67	29.52	31.18	25.38
Chlorine (Cl)	17	35.5	0.60	0.22	--	--

## Data Availability

Not applicable.
